# Gastro-BaseNet: A Specialized Pre-Trained Model for Enhanced Gastroscopic Data Classification and Diagnosis of Gastric Cancer and Ulcer

**DOI:** 10.3390/diagnostics14010075

**Published:** 2023-12-28

**Authors:** Gi Pyo Lee, Young Jae Kim, Dong Kyun Park, Yoon Jae Kim, Su Kyeong Han, Kwang Gi Kim

**Affiliations:** 1 Department of Health Sciences and Technology, Gachon Advanced Institute for Health Sciences and Technology (GAIHST), Gachon University, Incheon 21565, Republic of Korea; jeju5582@gmail.com; 2 Department of Biomedical Engineering, Gachon University Gil Medical Center, College of Medicine, Gachon University, Incheon 21565, Republic of Korea; kimyj10528@gmail.com; 3 Division of Gastroenterology, Department of Internal Medicine, Gachon University Gil Medical Center, College of Medicine, Gachon University, Incheon 21565, Republic of Korea; pdk66@gilhospital.com (D.K.P.); yoonjaemed@gmail.com (Y.J.K.); 4 Health IT Research Center, Gachon University Gil Medical Center, Incheon 21565, Republic of Korea; skhan1211@naver.com

**Keywords:** gastroscopy, transfer learning, deep learning, Gastro-BaseNet, ImageNet, endoscopy

## Abstract

Most of the development of gastric disease prediction models has utilized pre-trained models from natural data, such as ImageNet, which lack knowledge of medical domains. This study proposes Gastro-BaseNet, a classification model trained using gastroscopic image data for abnormal gastric lesions. To prove performance, we compared transfer-learning based on two pre-trained models (Gastro-BaseNet and ImageNet) and two training methods (freeze and fine-tune modes). The effectiveness was verified in terms of classification at the image-level and patient-level, as well as the localization performance of lesions. The development of Gastro-BaseNet had demonstrated superior transfer learning performance compared to random weight settings in ImageNet. When developing a model for predicting the diagnosis of gastric cancer and gastric ulcers, the transfer-learned model based on Gastro-BaseNet outperformed that based on ImageNet. Furthermore, the model’s performance was highest when fine-tuning the entire layer in the fine-tune mode. Additionally, the trained model was based on Gastro-BaseNet, which showed higher localization performance, which confirmed its accurate detection and classification of lesions in specific locations. This study represents a notable advancement in the development of image analysis models within the medical field, resulting in improved diagnostic predictive accuracy and aiding in making more informed clinical decisions in gastrointestinal endoscopy.

## 1. Introduction

According to 2020 GLOBOCAN statistics, gastric cancer is the most common malignancy among men and women, with more than 1 million new cases worldwide and the fifth highest incidence (5.6%) and fourth highest mortality (7.7%) among men and women combined. The highest incidence of gastric cancer in the world is in East Asia and Eastern Europe [1]. Gastric cancer is diagnosed through endoscopy, which visually confirms the location and type of tumor in the stomach. Histologic confirmation through biopsy is necessary to make a final diagnosis [2]. However, gastroscopy misses gastric cancer between 4.6% and 14.3% of the time because it requires the examiner to detect the lesion with the naked eye [3].

Recently, researchers have been applying artificial intelligence (AI) and convolutional neural networks (CNN) to detect advanced gastric cancer and early gastric cancer. Lee et al. [4] developed a malignancy prediction model with an accuracy of 96.49% (normal and gastric cancer classification) and 77.12% (gastric cancer and gastric ulcer) using CNN-based models of ResNet50, InceptionV3, and VGG16. Ma et al. [5] developed an early gastric cancer classification model with an accuracy of 98.84% and an F1 score of 98.18% using GAIN-ResNet50 using attention maps. Wei et al. [6] developed a DCNN model using VGG16 and ResNet50 to classify non-malignant mucosa (normal) and gastric cancer, with an accuracy of 92.50% and a sensitivity of 94%. In particular, both senior and novice endoscopists showed higher accuracy and performance. Teramoto et al. [7] developed a gastric cancer prediction model based on VGG, DenseNet, InceptionV3, and ResNet; the DenseNet121 model showed the highest performance with a sensitivity of 97.0% and a specificity of 99.4%. Yuan et al. [8] trained a diagnostic model using the YOLO architecture to determine the most predicted results as the final diagnosis, with a classification accuracy of 93.5%, sensitivity of 59.2%, and specificity of 99.3% for early gastric cancer and a classification accuracy of 98.4%, sensitivity of 100ss%, and specificity of 98.1% for advanced gastric cancer. Cho et al. [9] developed a binary classification model to classify cancer and non-cancer using InceptionResNetV2, with an accuracy of 76% and an AUC of 0.706.

Those previous studies developed a CNN model for detecting and predicting the diagnosis of both early and advanced stages of gastric cancer. This did not involve training a new model with randomly assigned weight values. Instead, a pre-trained model was fine-tuned on a large dataset using the weights of the initial model, referred to as transfer learning. The widely used dataset for pretraining models was ImageNet [10], and its weights were utilized during the transfer learning process. However, ImageNet was an image dataset of a thousand classes for various objects and was pre-trained with images from a completely different environment than gastroscopy images. This study presented the development of a pre-trained model called Gastro-BaseNet, which utilized gastroscopic images to classify normal and abnormal diagnoses, such as gastric cancer and ulcers. During the pre-training phase, Gastro-BaseNet acquired information about the characteristics of abnormal gastric lesions as opposed to those found in a normal stomach. This pre-trained knowledge in endoscopic images formed the basis of our pre-trained information and enhanced training efficiency compared to other pre-trained models such as ImageNet, which relied on knowledge from other natural image domains.

Our study involved the development of Gastro-BaseNet through the training of model layers and weights, specifically for the classification of abnormal gastric diseases. This model proved valuable in predicting specific gastric diagnoses and replaced pre-trained models in more specialized studies that involve gastroscopic images. Therefore, we utilized Gastro-BaseNet to classify gastric cancer and ulcers, comparing its performance with a predictive model that employed the weights of pre-trained models to confirm the feasibility of using an optimized model for research on endoscopic datasets.

## 2. Materials and Methods

### 2.1. Data Acquation

We constructed a training database for Gastro-BaseNet to predict abnormal gastric lesions utilizing gastroscopic image data. We had examined the medical and pathology records of patients who visited Gachon University Gil Hospital (IRB No. GBIRB 2021-383) between January 2019 and December 2022 and underwent gastroscopy to establish their final diagnosis (Table 1). We selected a total of 1902 patients, 1135 men and 767 women, with a mean age of 68.03 (±12.00), and divided into three categories: 1070 patients diagnosed with gastric cancer, 720 men and 350 women, with a mean age of 70.45 (±11.05), 532 patients diagnosed with gastric ulcer, 317 men and 215 women, with a mean age of 67.19 (±12.61), and 300 patients with normal patients with no abnormal findings, 98 men and 202 women, with a mean age of 60.86 (±11.09), based on the pathology results. The images collected from patients consisted of frozen images manually captured from the screen during gastroscopy by a gastrointestinal specialist. For patients with a diagnosis, only images containing regions of cancer or ulcer were collected, while for normal patients, all images taken during the examination were collected. Therefore, the number of images collected for each patient varies by disease. The average number of frozen images collected per patient, by diagnosis, was approximately 5.76 (±3.48) of gastric cancer, 6.20 (±4.15) of ulcers, and 33.52 (±10.65) of normal cases. Finally, the dataset consisted of a total of 19,518 images and consisted of 6164 images of gastric cancer, 3297 images of gastric ulcers, and 10,057 images of normal regions.

Then, we constructed another dataset to verify the performance of transfer learning by Gastro-BaseNet. We collected still images of patients diagnosed with gastric cancer and gastric ulcers through histologic examination from June 2018 to November 2021 who visited Gachon University Gil Hospital. When selecting the patient’s data for evaluating transfer learning according to a model pre-trained by gastroscopic data, the patients collected for training Gastro-BaseNet were excluded. To evaluate the efficiency of the transfer learning on Gastro-BaseNet, additional studies were conducted to develop diagnostic models for two types of gastric lesions: gastric cancer (study 1) and gastric ulcers (study 2). In Table 2, we selected a total of 1033 patients, 660 men and 373 women, with a mean age of 65.56 (±13.00), and divided them into three categories: 707 patients diagnosed with gastric cancer used for study 1484 men and 223 women, with a mean age of 68.01 (±11.82), 178 patients diagnosed with gastric ulcer used for study 2121 men and 57 women, with a mean age of 62.45 (±14.52), and 148 patients with normal patients that had nothing abnormal findings, 55 men and 93 women, with a mean age of 57.62 (±12.56), based on the pathology results. The average number of frozen images collected per patient, by diagnosis, was approximately 5.27 (±3.09) of gastric cancer, 6.31 (±3.98) of gastric ulcers, and 34.82 (±11.24) of normal cases. Finally, the dataset consisted of a total of 10,001 images and consisted of 3724 images of gastric cancer, 1124 images of gastric ulcers, and 5153 images of normal regions.

All images were captured in the white-light imaging mode. The image was extracted into JPG format, and the resolution of the original image was very varied, ranging from 187 × 186 to 1350 × 1062 pixels.

### 2.2. Study Environment

The system environment for deep learning employed an IBM Power System AC922 8335-GTH (IBM, Armonk, NY, USA) with a single NVIDIA Tesla V100-SXM2 32 GB (NVIDIA, Santa Clara, CA, USA). The operating system used was Ubuntu 18.04.5. We used Python (version 3.7.10) with TensorFlow frameworks (version 2.6.0) and Keras (version 2.6.0) for training and evaluating models. For image processing, the OpenCV (version 4.6.0) libraries were used.

### 2.3. Data Preprocess

**Figure 1:**
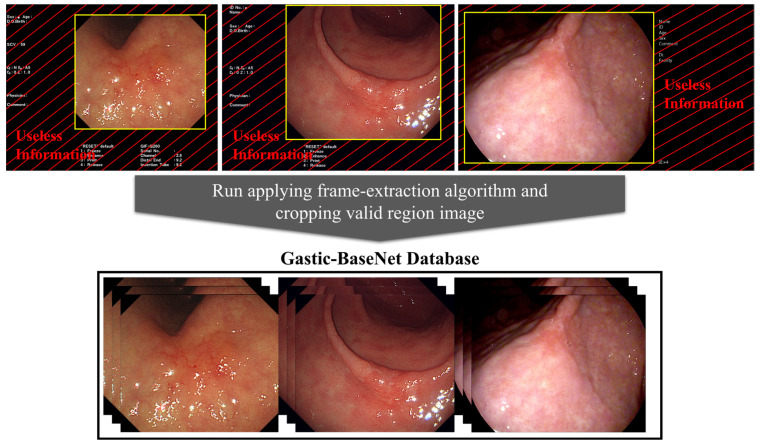
The process of cropping useless information regions from the initial collected gastroscopic images and building an image database.

Freeze image, captured screen in esophagogastroduodenoscopy, included useless information about patient name, patient ID, study date, endoscopy device, etc. These were located outside of the video frame in most of the images. To extract a valid area of gastroscopy video, we applied a frame-extraction algorithm and cropped the images of a valid region to train models from them (Figure 1). In a few images, some areas of marked information invaded the valid frame. However, these data included the training dataset because it improved the generality of the trained model as a result of noise and prevented the loss of clear cancer or ulcer areas.

To train deep learning models, we resized the collected images with the ImageNet dataset to the image size of the pre-trained models’ input shape. The pre-trained models used in this study were ResNet50 and EfficientNetB0, both with a resolution of 224 × 224.

### 2.4. Transfer Learning

Transfer learning is a training technique that transfers the processing ability of a pre-trained model to solve one task and utilizes it to solve another task [11,12]. By using a large amount of data, the feature extraction phase of a well-trained model is recalled and transferred to learn the new task. In this case, the training time is reduced, and the trained model’s performance is improved. Transfer learning trains the weights in two different ways, according to the users’ objectives [13]. One is called freeze mode, which freezes the feature extraction phase of the pre-trained model and allows only the new classifier part to be trained. This is used when the pre-trained model and the new training dataset have a similar problem or domain image. The other is called fine-tune mode through the pre-trained model, and the new training dataset has completely different problems or domain images; both feature extraction and classifiers are trained and tuned [14]. This can be carried out by training all layers of the feature extraction part, or by freezing some layers (lower layer part) that extract general features and training the rest of the layers (upper layer part) that extract problem-specific features.

**Figure 2:**
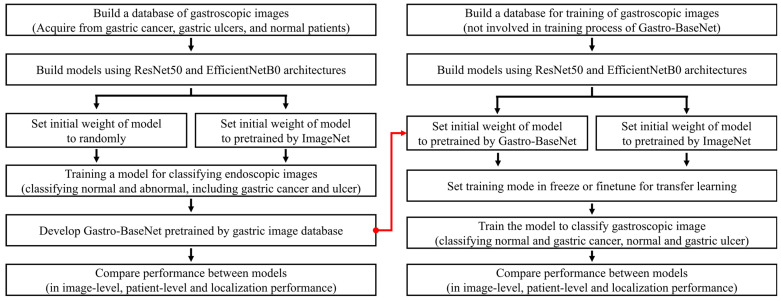
Flowchart of the process for developing Gastro-BaseNet, a pretraining model used for gastroscopic images, and validating the training results with the pretraining model; the red line means the developed pretrained Gastro-BaseNet was used for the initial weight setting.

For transfer learning, the fully connected (FC) layer responsible for the classifier of the existing trained CNN model is removed, and new FC layers are connected to adjust the weights through training for our purpose [15]. Gastro-BaseNet was used a pre-trained model from the ImageNet database to classify normal or abnormal (including gastric cancer and ulcers) in gastroscopic images to transfer learning. To develop a predictive model for gastric cancer or ulcer, we used two pre-trained models (ImageNet, which is generally used, and Gastro-BaseNet, which is trained by a gastroscopic database) and compared their performance (Figure 2).

In this study, ResNet50 [16] and EfficientNetB0 [17] were used as base architectures. These CNN models were built with a base architecture and applied with pre-trained weights open to the public. These were built by removing the existing FC layers and adding layers for global average pooling, batch normalization, and a new FC layer for binary classification. Models were fine-tuned to predict gastric diseases using a newly constructed training and validation dataset.

### 2.5. Setting Model Training Hyperparameters

**Figure 3:**
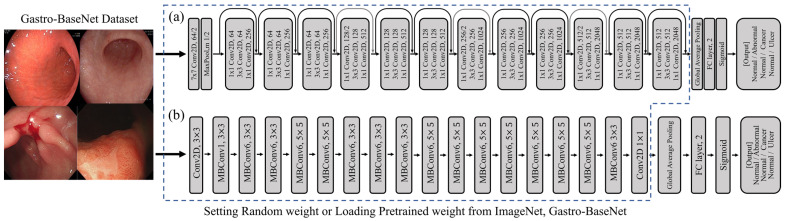
The architectures of the models used for deep learning training are (**a**) ResNet50 and (**b**) EfficientNetB0.

Pre-trained models used for diagnosing gastric cancer or gastric ulcers were trained based on ResNet50 and EfficientNetB0 architectures (Figure 3). The layers from model architectures and the weight were loaded, and the top block that functions as a classifier was removed. The training model was built by connecting the feature extraction part, excluding the removed block, and a new classifier block for binary classification of normal and gastric lesions. The new classifier block consisted of global average pooling, a batch normalization layer, and an FC layer for predicting the final classified result. For the training model using transfer learning, two model training methods were used: fine-tune mode, which adjusts the weights of all layers, and freeze mode, which adjusts the weights of the classifier only. The hyperparameters for training were set to stochastic gradient descent (SGD), momentum 0.9, decay 0.0001, learning rate 0.0001, batch size 256, and epoch 1000, with an early stopping setting to terminate training if learning did not improve in 60 epochs. All model training was performed with the same settings. For training models using transfer learning, two model training methods were used: fine-tune mode, which adjusts the weights of all layers, and freeze mode, which adjusts the weights of the classifier only. The hyperparameters for training were set to stochastic gradient descent (SGD), momentum 0.9, decay 0.0001, learning rate 0.0001, batch size 256, and epoch 1000, with an early stopping setting to terminate training if learning did not improve in 60 epochs. All model training was performed with the same settings.

### 2.6. Statistical Analysis

#### 2.6.1. Image-Level Performance

To evaluate the performance of the trained CNN model based on Gastro-BaseNet, performance metrics were calculated and compared with the results obtained with ImageNet. A new, unseen image (not involved at all in the training stage) was applied to the training model to predict whether the image was a gastric cancer or a gastric ulcer for each purpose. The predicted diagnosis was compared to the label (ground truth, GT) and represented according to a confusion matrix. Based on indications including true positive (TP), true negative (TN), false positive (FP), and false negative (FN) calculated by the confusion matrix, classification performance indicators with accuracy, sensitivity, specificity, and F1 score were calculated. Receiver operating characteristic (ROC) analysis [18] and area under the curve (AUC) were conducted to compare transfer-learned performance based on Gastro-BaseNet and ImageNet.

#### 2.6.2. Patient-Level Performance

The collected image dataset was built by acquiring multiple frozen images from a single patient. However, the performance evaluation at the image level performed earlier calculated performance indications for every result as an independent event, even if they were the same lesions from the same patient.

Thus, we conducted patient-level analysis, and the predicted diagnosis result with the largest number of images diagnosed by the AI model was regarded as the final diagnosis for that patient case. At this time, if the number of counts for each decision diagnosed as normal or abnormal by the AI system was the same in the same patient, the patient’s final diagnosis was considered undetermined and excluded from the evaluation metric calculation.

#### 2.6.3. Localization Performance

Grad-CAM is a proposed method for explainable AI that uses a heatmap to represent weights to explain what the model is looking at and making decisions about [19]. Grad-CAM extracted from the model classifying as diagnosis highlighted the areas in red color with a high probability of localizing a lesion. Using this, a thresholding algorithm can be applied to specify the location of the lesion as predicted by the AI model.

**Figure 4:**
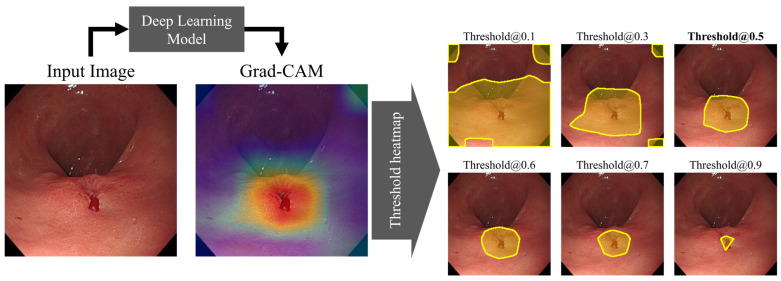
The evaluation process for calculating localization performance based on Grad-CAM; yellow line: the location of the lesion predicted by the model, the bolded threshold: the value used for localization in this study.

In this study, we set a threshold value for localization to 0.5 in grad-CAM and set the area above the threshold value as the location of the lesion predicted by the model. We calculated the sensitivity of the detected location by comparing the interaction over union (IoU) score with the lesion location manually labeled and determining the case above 0.3 of the IoU as TP. These processes were introduced in Figure 4.

## 3. Results

### 3.1. Gastro-BaseNet

**Figure 5:**
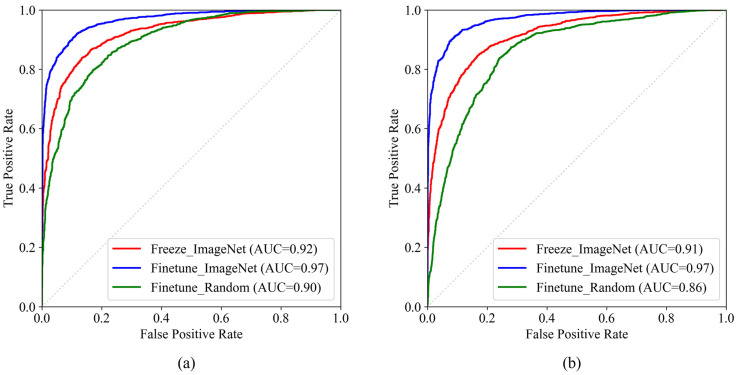
Comparison of ROC curves for normal and abnormal classification models: (**a**) comparison of model performance by training method based on ResNet50 architecture. (**b**) comparison of model performance by training method based on EfficientNetB0 architecture.

We developed a pre-trained model, Gastro-BaseNet, based on gastroscopic images. Gastro-BaseNet was trained to classify and predicted normal cases and abnormal cases, including stomach cancer and stomach ulcers. We developed Gastro-BaseNet by comparing the performance of the model trained by setting the initial weight randomly and the pre-trained weight with ImageNet as the initial weight, based on the ResNet50 and EfficientNetB0 model architectures (Table 3). In the two models using ResNet50 and EfficientNetB0, the model trained by tuning the weights of the entire network through fine-tune mode from the pre-trained weight as the ImageNet dataset showed the highest performances with accuracy of 91.88% and 91.93% and sensitivity of 90.38% and 91.26% in image-level performance. Then it showed accuracy of 95.42% and 95.92% in each model architecture in patient-level performance and the highest sensitivity performance of 82.19% in localization performance when trained with the ResNet50 architecture. The ROC analysis and pairwise comparison of ROC curves were conducted on the predicted performance of the model. Figure 5 showed the comparison of ROC curves for two model architectures. In both cases, models trained through fine-tune mode based on ImageNet pre-trained models exhibited the highest performance with AUCs of 96.54% and 96.95%, and the differences were statistically significant compared to training from random initial weights and freeze mode. The *p*-values for all results of the pairwise comparison of ROC curves was lower than 0.0001.

### 3.2. Models Trained on Transfer Learning Based on Gastro-BaseNet

We used a gastroscopic dataset to develop a model to predict normal and abnormal diagnosis, including gastric cancer and gastric ulcers. Using the developed Gastro-BaseNet for classifying abnormal diagnosis as a pre-trained weight, we applied transfer learning techniques to develop training models to predict the diagnosis of gastric cancer and gastric ulcer, respectively. We compared the performance differences of the training models with the traditional ImageNet weights.

#### 3.2.1. Gastric Cancer Classification Trained by Gastro-BaseNet

**Figure 6:**
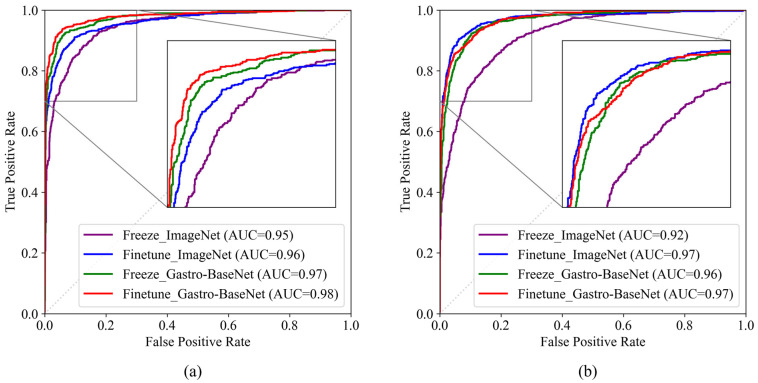
Comparison of ROC curves for normal and gastric cancer classification models using Gastro-BaseNet: (**a**) comparison of model performance by training method based on ResNet50 architecture; (**b**) comparison of model performance by training method based on EfficientNetB0 architecture.

We developed classification models that predict the presence of gastric cancer disease in input images of endoscopy through transfer learning based on Gastro-BaseNet. Except for the pre-trained model for transfer learning, the rest of the training environment was set the same. In training using the ResNet50 architecture, the performance of the model with transfer learning using Gastro-BaseNet was 94.07% in freeze mode and 94.72% in fine-tune mode, and the accuracy was improved by 3.61% and 4.05% compared to the performance of the model with transfer learning using ImageNet in the same training mode (Table 4). However, in training with the EfficientNetB0 architecture, the performance of the model trained with ImageNet showed higher accuracy than Gastro-BaseNet. Nevertheless, the sensitivity performance of the model using Gastro-BaseNet is 98.97%, which is an improvement of 10.39% and 5.64% in each mode compared to that using ImageNet, and the AUC score of the model using Gastro-BaseNet is improved by 3.06% when trained in freeze mode for image-level performance. The results of the ROC comparison analysis using the ResNet50 architecture are presented in Figure 6a. Transfer learning using Gastro-BaseNet with the ResNet50 architecture demonstrated the highest performance, with AUC scores of 97.90% and 97.43% in fine-tune and freeze training modes, respectively. According to Table 5, there was a statistically significant difference with training by ImageNet in both freeze and fine-tune modes (*p*-values were lower than 0.0001). Figure 6b shows the ROC curves using the EfficientNetB0 architecture. The model performance using Gastro-BaseNet in fine-tune mode showed an AUC of 96.42%. The result of the pairwise comparison of ROC curves in Table 5 between Gastro-BaseNet and ImageNet in freeze mode revealed a *p*-value lower than 0.0001, indicating a statistically significant difference.

In patient-level performance, the model using ResNet50 architecture-based Gastro-BaseNet showed higher accuracy and sensitivity than ImageNet. In the EfficientNetB0 model, the accuracy of the model using Gastro-BaseNet increased by more than 10%, and the sensitivity was higher than that of using ImageNet. In addition, the localization performance shows that the model using transfer learning with Gastro-BaseNet has superior location detection performance.

#### 3.2.2. Gastric Ulcer Classification Trained by Gastro-BaseNet

**Figure 7:**
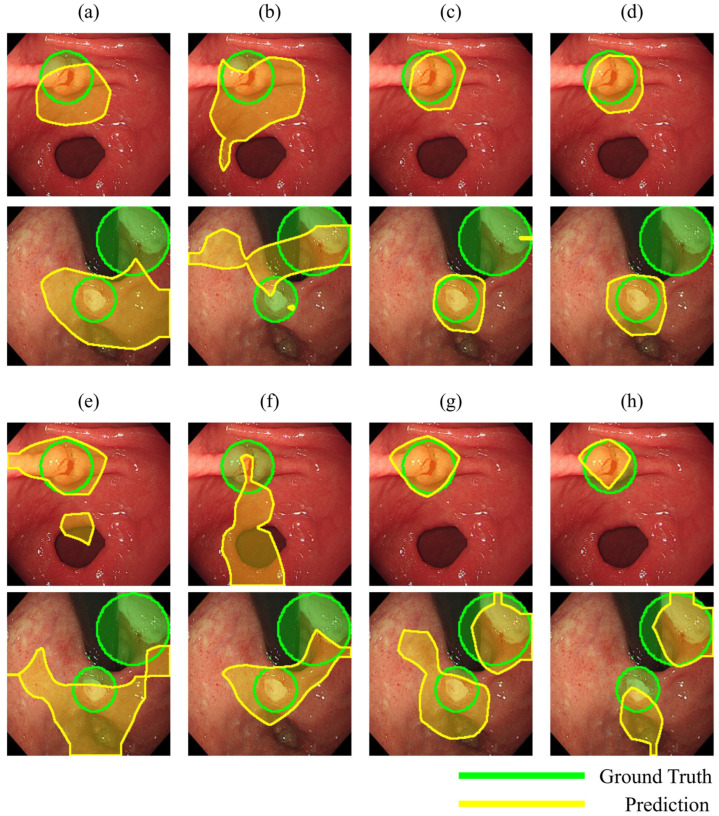
Results of evaluating localization performance based on Grad-CAM; transfer-learned on ResNet50 architecture (**a**) by ImageNet and freeze mode; (**b**) by ImageNet and fine-tune mode; (**c**) by Gastro-BaseNet and freeze mode; (**d**) by Gastro-BaseNet and fine-tune mode and transfer-learned on EfficientNetB0 architecture; (**e**) by ImageNet and freeze mode; (**f**) by ImageNet and fine-tune mode; (**g**) by Gastro-BaseNet and freeze mode; (**h**) by Gastro-BaseNet and fine-tune mode.

To develop a deep learning model to diagnose gastric ulcers, we trained the model by transferring weights based on Gastro-BaseNet. In terms of image-level performance (Table 6), the ResNet50 architecture was used to improve the accuracy from 92.03% to 92.72% and the F1 score of 95.14% and 95.62% in two learning modes using Gastro-BaseNet as pre-trained weights compared to ImageNet weights, showing an increase of about 4% and a 2.5% increase in performance. The highest accuracy of 92.31% and sensitivity of 85.71% were achieved in training in freeze mode using Gastro-BaseNet on patient-level performance. In terms of localization performance, training in freeze mode using Gastro-BaseNet showed the highest detection accuracy, with a detection sensitivity of 90.80%. In image-level performance using the EfficientNetB0 architecture, the learning performance of the model using ImageNet weights was higher than that of the model using Gastro-BaseNet, with 88.54% and 88.62% of accuracy and 93.06% and 93.11% of F1 scores. However, in other performances about the diagnosis of gastric ulcer, the AUC scores of the model using Gastro-BaseNet were 90.84% and 90.04%, which showed higher performance than the model using ImageNet, especially in patient-level performance, with higher accuracy of 89.39% and 88.89%. In terms of localization performance, the freeze training mode using Gastro-BaseNet showed 81.82% localization performance. Figure 7 shows the results of predicting the location of lesions extracted by grad-CAM and manually labeling the lesion location as GT. Compared to ImageNet, the model trained on Gastro-BaseNet focused on detecting regions that were very similar to the GT regions. In particular, the model trained on EfficientNetB0 found similar locations for two lesions that were missed by all other training models.

**Figure 8:**
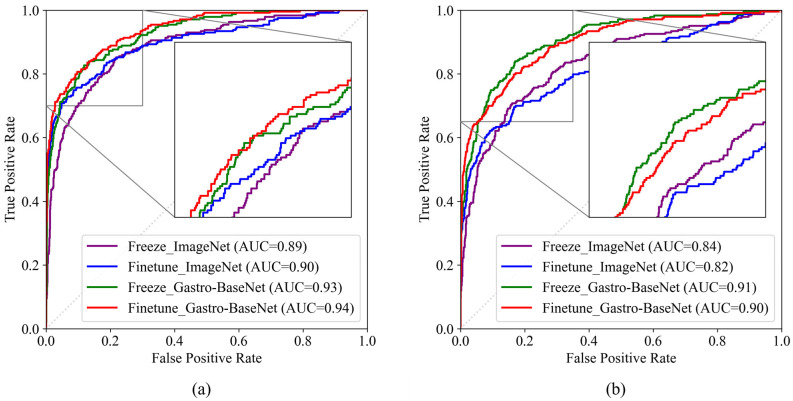
Comparison of ROC curves for normal and gastric ulcer classification models using Gastro-BaseNet: (**a**) comparison of model performance by training method based on ResNet50 architecture; (**b**) comparison of model performance by training method based on EfficientNetB0 architecture.

In the ResNet50 architecture, the highest AUC score of 93.82% was achieved using Gastro-BaseNet in fine-tune mode, as shown in Figure 8a. According to the pairwise comparison results of the ROC curves in Table 7, there is a *p*-value of 0.0005 in the freeze mode and a *p*-value of 0.0004 in the fine-tune mode. While in the EfficientNetB0 architecture (Figure 8b), both training modes showed a *p*-value lower than 0.0001 in pairwise comparison results, indicating a significant difference in the training using Gastro-BaseNet.

## 4. Discussion

In this study, we developed a pre-trained model using gastroscopic data and used it to train a deep learning model to predict gastric cancer or gastric ulcer; these compared the performance of the model trained with ImageNet, which is widely used [20]. Then, it generated Gastro-BaseNet, a model more optimized for gastroscopic images. These showed higher performance than the model performance that was transfer-learned by ImageNet. To develop a pre-trained model using gastroscopic data, we trained classification on images of abnormal lesions, including gastric cancer and gastric ulcer. To demonstrate the training effectiveness in general transfer learning, some experiments were conducted: first, we trained the initial model weights set at random. Second, the model was trained in freeze mode, using the weights of the pre-trained model like ImageNet to train only a part of the classifier. At last, the model was trained in fine-tune mode, and the entire network was tuned with small changes. To verify the performance of the trained model, we fed the model with a test dataset that was not involved in training and compared the prediction results. Two evaluation metrics were introduced and compared: image-level, which evaluates the total number of images as independent cases, and patient-level, which extracts individual results for images collected from the same patient and evaluates the most frequent prediction as the final result. Additionally, we compared the localization performance based on the location information extracted using grad-CAM, which was generated from models trained with abnormal data containing gastric lesions.

The pre-trained model to classify the abnormal classes of gastroscopic images showed an accuracy of 91.88% and 91.93% in the studies using ResNet50 and Efficient-NetB0, respectively, using transfer learning in fine-tune mode based on ImageNet, with AUC scores of 96.54% and 96.95%, and a sensitivity to localization of 82.19% in ResNet50 and 70.74% in EfficientNetB0 for very high performance in classification and localization. This indicates that it has been learned to identify the features of gastric cancer or gastric ulcer at the correct location. This confirms that models that were trained by ImageNet performed faster and better than learning weights from random models, despite training endoscopic information from completely different domains.

Next, we developed a diagnostic prediction model for gastric cancer or ulcer through transfer learning using Gastro-BaseNet. The model trained on a dataset with the domain of gastroscopy based on Gastro-BaseNet proved the prediction performance of using a pre-trained model that trained on images with a similar domain by comparing the results of transfer learning with ImageNet.

In the training of the gastric cancer classification model, the transfer learning method in fine-tune mode using Gastro-BaseNet based on the ResNet50 architecture showed the highest performance with 94.72% of accuracy, 94.10% of sensitivity, and an AUC score of 97.90%. Additionally, the localization performance was also excellent, with a sensitivity to localization 87.19%. In the training based on the EfficientNetB0 architecture, transfer learning using Gastro-BaseNet showed lower specificity compared to ImageNet (94.51% and 72.19%, respectively, in fine-tune mode). However, the sensitivity for classification of gastric cancer was the highest at 98.97% in fine-tune mode training using Gastro-BaseNet, and the accuracy was 95.88% in patient-level performance evaluation. Furthermore, performance in localization was 78.50%, which was 10.04% higher than ImageNet. In the classification model for gastric ulcers, the performance of the model using Gastro-BaseNet in the ResNet50 architecture was the highest, with an accuracy of 92.72%, an F1 score of 95.62%, and an AUC score of.93.82%. In the localization performance, especially, the sensitivity to localization in fine-tune mode based on Gastro-BaseNet showed a very high indication at 90.80%. Also, similarly to the gastric cancer classification model trained on the EfficientNetB0 architecture, it had lower accuracy and specificity compared to ImageNet. Nevertheless, it showed high performance at a sensitivity of 95.06% in freeze mode, and localization performance also showed a high sensitivity of 81.82%.

Until now, most of the training methods for developing deep learning models based on medical images (especially classification training for diagnostic prediction) have been performed based on transfer learning. The pre-trained model used at this time brought up and used the weight values of the model using ImageNet, and it showed excellent performance [14,21]. However, ImageNet-based pre-trained models did not have prior learning knowledge of medical domains. Studies about pre-trained models based on data from medical domains were not active due to many limitations on data collection, personal information protection, etc. Therefore, our study created a pre-trained model with knowledge gained through collecting various patients and images and learning about gastroscopy data that trained them, named Gastro-BaseNet.

The first limitation of our study is that we set the entire layer to be trained without proper adjustment over the number of trainable layers in the pre-trained model. The trans-fer learning may adjust the number of layers to be trained according to the domain of the training data of the pre-trained model and the domain of the training data of the newly trained model. In addition, some studies have stated that transfer learning using natural data such as ImageNet on medical data only speeds up the convergence of learning and does not affect performance improvement [22,23,24]. In this study, the performance improvement of the new learning model was demonstrated by a model pre-trained with gastroscopy data compared to the existing ImageNet, but it showed very high sensitivity in training based on the EfficientNetB0 model but also reduced accuracy and specificity performance.

The second limitation of our study is that we trained only on two kinds of model architectures. It may lead to a lack of diversity and limitations for use in various studies that can be conducted later. Also, we developed a pre-trained model using data collected from one medical institution and identified a diagnostic prediction model using it. Gastro-BaseNet used more than 10k images, but the diversity may be somewhat reduced due to the inclusion of many images taken from the same patient. This resulted in higher predictive performance than ImageNet without prior knowledge of the medical domain, but the lack of generalization of the model may lead to poor classification performance for collected gastroscopic images in new environments (other endoscopic devices, imaging systems, etc.).

The third limitation of our study is that we have learned the deep learning model only from the collected data without obtaining and performing more training data through data augmentation [25,26]. Deep learning requires a large amount of data, and in general, the more learning data, the higher the training performance. Although we have collected and trained large amounts of data compared to other previous studies, we can achieve performance improvements by training additional data together through image processing techniques or generative adversarial network techniques [27], etc.

The fourth limitation of our study is the lack of a more detailed analysis of the evaluation of localization. Generally, specialized architectural models for detection, such as YOLO [28] and SSD [29], are employed to predict the location of gastric lesions, offering the advantage of real-time prediction [30]. In this study, the evaluation of the localization level involved calculating the sensitivity performance for detection based on the IoU value. This can serve as a detection algorithm because the Gastro-BaseNet-based transfer-learned model demonstrates a significant difference compared to other trained models. For a quantitative comparison with results based on existing detection models, an analysis of new performance indicators such as mean average precision (mAP) and frames per second (FPS) is required. However, when using the classification model, extracting detection results through Grad-CAM involves additional steps. Consequently, we anticipate that the FPS performance, indicating the rate at which detection results are obtained, will differ significantly compared to typical detection models.

In future studies, we will secure various pre-trained models using gastroscopic image domains through training using more CNN model architectures. The secured pre-trained model will be widely used not only for various gastroscopy-related studies in the future but also for training various endoscopic medical data such as colonoscopy and esophageal endoscopy. In addition, through multicenter research, we want to build an image database captured and stored in various environments and improve the generality of the deep learning model through additional studies using it. In addition, studies are being actively conducted to develop a deep learning model that combines machine learning to predict models through selected features by applying feature fusion and selection algorithms based on extracted image features rather than classifier methods through FC layers [31]. We will explore various ensemble techniques and machine learning algorithms combined to enhance model performance and aim to compare and validate the results obtained through the application of transfer learning.

## 5. Conclusions

This study represents a significant stride forward in the development of diagnostic prediction fields for gastric diseases by developing pre-trained models specialized in medical image domains, especially gastroscopic images, rather than conventional natural data for deep learning models. Additionally, models pre-trained on data from a similar domain have exhibited substantial improvements in their localization performance on objects, indicating a high potential for use as a detection algorithm. Specifically, it implies that a state-of-the-art classification learning model based on the transformer algorithm, such as Vision Transformer (ViT) [32] or DeiT [33], has the capability to perform detection concurrently. The results verified through our studies will accelerate the development of advanced deep learning techniques that can be utilized in various endoscopic image-based artificial intelligence areas. With the recent advent of foundation models [34], transfer learning has emerged as an approach to improve training efficiency and reduce data requirements. This study attempts to emphasize the need to develop more specialized pre-trained models or foundation models. The final goal is to contribute to the improvement of predictive capabilities for the diagnosis of gastric diseases, enabling direct use in the actual clinical environment and paving the way for the future development of artificial intelligence technology in the field of gastrointestinal endoscopy as an early diagnosis and assistance system.

## Figures and Tables

**Table 1:** Number of datasets built for training Gastro-BaseNet and datasets for deep learning training.

	Normal	Abnormal		Total
		Gastric Cancer	Gastric Ulcer	
Train	7297	4476	2409	14,182
Validation	738	493	263	1494
Test	2022	1195	625	3842
Total (Number of patients)	10,057 (300)	6164 (1070)	3297 (532)	19,518 (1902)

**Table 2:** Number of patients and image datasets for training models to classify gastric cancers (study 1) or gastric ulcers (study 2) by using transfer learning based on Gastro-BaseNet.

	Study 1: Gastric Cancer			Study 2: Gastric Ulcer		
	Normal	Gastric Cancer	Total	Normal	Gastric Ulcer	Total
Train	3662	2671	6333	3617	789	4406
Validation	416	273	689	461	92	553
Test	1075	780	1855	1075	243	1318
Total (Number of patients)	5153 (148)	3724 (707)	8877 (855)	5153 (148)	1124 (178)	6277 (326)

**Table 3:** Result of Gastro-BaseNet performance, a classification model for abnormalities indicative of gastric lesions, by pretrained weights and training mode.

Model Architecture	Hyperparameters		Image-Level Performance (%)					Patient-Level Performance (%)			Localization Performance (%)
	Training Mode	Pretrained Weight	Accuracy	Sensitivity	Specificity	F1 Score	AUC	Accuracy	Sensitivity	Specificity	Sensitivity
ResNet50	fine-tune	Random	83.91	87.42	80.76	84.09	89.57	88.38	90.06	79.31	41.23
	freeze	ImageNet	88.50	87.09	89.76	89.15	92.39	92.68	91.61	98.31	58.11
	fine-tune	ImageNet	91.88	90.38	93.22	92.36	96.54	95.42	94.53	100.0	82.19
EfficientNetB0	fine-tune	Random	79.54	84.29	75.27	79.48	86.10	83.11	85.94	68.33	63.43
	freeze	ImageNet	87.79	87.25	88.28	88.39	91.44	92.43	91.94	95.00	61.52
	fine-tune	ImageNet	91.93	91.26	92.53	92.35	96.95	95.92	95.13	100.0	70.74

**Table 4:** Results of gastric cancer classification model performance trained by Gastro-BaseNet and ImageNet by pretrained weights and training mode.

Model Architecture	Hyperparameters		Image-Level Performance (%)					Patient-Level Performance (%)			Localization Performance (%)
	Training Mode	Pretrained Weight	Accuracy	Sensitivity	Specificity	F1 Score	AUC	Accuracy	Sensitivity	Specificity	Sensitivity
ResNet50	freeze	ImageNet	90.46	90.26	90.60	91.67	94.81	96.99	96.32	100.0	80.26
	fine-tune	ImageNet	90.67	92.44	89.40	91.74	96.22	95.29	94.29	100.0	82.94
	freeze	Gastro-BaseNet	94.07	93.72	94.33	94.86	97.43	97.66	97.16	100.0	83.99
	fine-tune	Gastro-BaseNet	94.72	94.10	95.16	95.43	97.90	97.66	97.16	100.0	87.19
EfficientNetB0	freeze	ImageNet	88.79	87.82	89.49	90.24	91.73	92.26	91.37	96.55	67.15
	fine-tune	ImageNet	94.02	93.33	94.51	94.82	97.05	97.66	97.16	100.0	68.54
	freeze	Gastro-BaseNet	85.71	98.21	76.65	86.15	97.01	95.32	99.29	7667	75.72
	fine-tune	Gastro-BaseNet	83.45	98.97	72.19	83.49	96.42	95.88	100.0	7667	78.50

**Table 5:** Results of pairwise comparison of ROC curves of the gastric cancer classification model trained by Gastro-BaseNet and ImageNet by pretrained weights and training mode.

Model Architecture	Variable 1		Variable 2		*p*-Value
	Training Mode	Pretrained Weight	Training Mode	Pretrained Weight	
ResNet50	ImageNet	freeze	ImageNet	fine-tune	0.0006
	ImageNet	freeze	Gastro-BaseNet	freeze	<0.0001
	ImageNet	freeze	Gastro-BaseNet	fine-tune	<0.0001
	ImageNet	fine-tune	Gastro-BaseNet	freeze	0.0002
	ImageNet	fine-tune	Gastro-BaseNet	fine-tune	<0.0001
	Gastro-BaseNet	fine-tune	Gastro-BaseNet	freeze	<0.0001
EfficientNetB0	ImageNet	freeze	ImageNet	fine-tune	<0.0001
	ImageNet	freeze	Gastro-BaseNet	freeze	<0.0001
	ImageNet	freeze	Gastro-BaseNet	fine-tune	<0.0001
	ImageNet	fine-tune	Gastro-BaseNet	freeze	0.0981
	ImageNet	fine-tune	Gastro-BaseNet	fine-tune	0.8909
	Gastro-BaseNet	fine-tune	Gastro-BaseNet	freeze	0.0270

**Table 6:** Results of gastric ulcer classification model performance trained by Gastro-BaseNet and ImageNet by pretrained weights and training mode.

Model Architecture	Hyperparameters		Image-Level Performance (%)					Patient-Level Performance (%)			Localization Performance (%)
	Training Mode	Pretrained Weight	Accuracy	Sensitivity	Specificity	F1 Score	AUC	Accuracy	Sensitivity	Specificity	Sensitivity
ResNet50	freeze	ImageNet	88.24	71.19	92.09	92.74	88.95	87.50	76.47	100.0	68.21
	fine-tune	ImageNet	88.54	63.79	94.14	93.06	88.90	87.50	76.47	100.0	64.52
	freeze	Gastro-BaseNet	92.03	76.54	95.53	95.14	92.76	92.31	85.71	100.0	80.11
	fine-tune	Gastro-BaseNet	92.72	71.60	97.49	95.62	93.82	88.89	78.79	100.0	90.80
EfficientNetB0	freeze	ImageNet	88.54	63.79	94.14	93.06	83.65	87.50	76.47	100.0	64.52
	fine-tune	ImageNet	88.62	63.79	94.23	93.11	81.97	85.71	72.73	100.0	72.90
	freeze	Gastro-BaseNet	74.51	95.06	69.86	81.72	90.84	89.39	100.0	76.67	81.82
	fine-tune	Gastro-BaseNet	83.76	76.54	85.40	89.56	90.04	88.89	78.79	100.0	68.28

**Table 7:** Results of pairwise comparison of ROC curves of the gastric ulcer classification model trained by Gastro-BaseNet and ImageNet by pretrained weights and training mode.

Model Architecture	Variable 1		Variable 2		*p*-Value
	Training Mode	Pretrained Weight	Training Mode	Pretrained Weight	
ResNet50	ImageNet	freeze	ImageNet	fine-tune	0.4423
	ImageNet	freeze	Gastro-BaseNet	freeze	0.0005
	ImageNet	freeze	Gastro-BaseNet	fine-tune	<0.0001
	ImageNet	fine-tune	Gastro-BaseNet	freeze	0.0165
	ImageNet	fine-tune	Gastro-BaseNet	fine-tune	0.0004
	Gastro-BaseNet	fine-tune	Gastro-BaseNet	freeze	0.0280
EfficientNetB0	ImageNet	freeze	ImageNet	fine-tune	0.3943
	ImageNet	freeze	Gastro-BaseNet	freeze	<0.0001
	ImageNet	freeze	Gastro-BaseNet	fine-tune	<0.0001
	ImageNet	fine-tune	Gastro-BaseNet	freeze	<0.0001
	ImageNet	fine-tune	Gastro-BaseNet	fine-tune	<0.0001
	Gastro-BaseNet	fine-tune	Gastro-BaseNet	freeze	0.4122

## Data Availability

The data are not publicly available due to restrictions, e.g., privacy or ethical.
